# Developing Dynamic Field Theory Architectures for Embodied Cognitive Systems with *cedar*

**DOI:** 10.3389/fnbot.2016.00014

**Published:** 2016-11-02

**Authors:** Oliver Lomp, Mathis Richter, Stephan K. U. Zibner, Gregor Schöner

**Affiliations:** ^1^Institut für Neuroinformatik, Ruhr-Universität Bochum, Bochum, Germany

**Keywords:** neural dynamics, dynamic field theory, artificial cognitive systems, autonomous robots, attractors, dynamic instabilities

## Abstract

Embodied artificial cognitive systems, such as autonomous robots or intelligent observers, connect cognitive processes to sensory and effector systems in real time. Prime candidates for such embodied intelligence are neurally inspired architectures. While components such as forward neural networks are well established, designing pervasively autonomous neural architectures remains a challenge. This includes the problem of tuning the parameters of such architectures so that they deliver specified functionality under variable environmental conditions and retain these functions as the architectures are expanded. The scaling and autonomy problems are solved, in part, by dynamic field theory (DFT), a theoretical framework for the neural grounding of sensorimotor and cognitive processes. In this paper, we address how to efficiently build DFT architectures that control embodied agents and how to tune their parameters so that the desired cognitive functions emerge while such agents are situated in real environments. In DFT architectures, dynamic neural fields or nodes are assigned dynamic regimes, that is, attractor states and their instabilities, from which cognitive function emerges. Tuning thus amounts to determining values of the dynamic parameters for which the components of a DFT architecture are in the specified dynamic regime under the appropriate environmental conditions. The process of tuning is facilitated by the software framework *cedar*, which provides a graphical interface to build and execute DFT architectures. It enables to change dynamic parameters online and visualize the activation states of any component while the agent is receiving sensory inputs in real time. Using a simple example, we take the reader through the workflow of conceiving of DFT architectures, implementing them on embodied agents, tuning their parameters, and assessing performance while the system is coupled to real sensory inputs.

## 1 Introduction

Neurally inspired architectures are a possible route along which artificial cognitive systems may be developed. However, designing and tuning neural architectures that generate intelligent behavior in embodied agents driven by real sensory inputs continues to be a challenge. While individual neural processing components, such as forward neural networks, can be tuned by learning, there is a gap between such neural processing and the capacity of an autonomous agent to organize its own behavior and cognitive processes under variable environmental conditions. In this paper, we address the problem of how autonomous embodied agents can be conceived, instantiated, and parameter tuned based on the principles of neural dynamics as formalized in *dynamic field theory* (DFT) (Schöner, 2008; Schöner et al., 2015b).

Neural dynamics, a subclass of neural network models pioneered by Grossberg (1978), combines the advantages of neural network thinking with the rigorous characterization of the functional properties of each computational element. Its modern variant, DFT, provides neural process accounts for behavior and cognition at the intermediate level of description of neural populations. Neural representations in DFT capture the continuous spatial, motor, or feature dimensions that are relevant to embodied, situated cognitive systems, avoiding the sampling of such dimensions by discrete neurons in conventional neural networks. This happens within neural fields that represent particular spatial locations, motor plans, or perceptual feature values by peaks of activation localized along these dimensions.

A core principle of DFT is the stability of meaningful activation patterns that are attractor states of the neural dynamics. The decision that a significant signal was detected in an input stream, for instance, is stabilized over a range of input strengths. Stability supports coupling of neural states to time-varying and noisy sensory input and enables neural dynamic models to act as controllers of effector systems.

Different attractor states represent different functional regimes of a DFT architecture. Each regime is delimited by characteristic instabilities that mediate qualitative change in neural representations as inputs vary, such as when working memory is first created, updated, or deleted. Designing a functional architecture in DFT entails specifying the conditions under which instabilities occur. Learning processes shift these conditions to new input configurations.

The tuning of parameters of DFT architectures focuses, therefore, primarily on assuring that within each neural field, the relevant instabilities occur when its inputs have the appropriate strength and form. When multiple neural fields are coupled, each field retains its functional properties as long as the attractors that instantiate these functions remain stable. This makes DFT architectures modular and enables them to scale.

The potential of DFT to provide scalable, modular neural dynamic architectures cannot be realized unless solutions are provided to the problems of designing complex architectures, parametrically tuning them, and evaluating their performance in closed loop with real environments. This paper analyzes these problems and provides solutions, captured by a modeling workflow and the software framework *cedar* (cognition, embodiment, dynamics, and autonomy in robotics).^1^

## 2 Concepts of Dynamic Field Theory

In this section, we briefly review the core concepts of dynamic field theory. We first introduce dynamic neural fields and nodes. Next, we discuss how these can be coupled to form architectures. Finally, we discuss how they may be connected to sensors and motor systems typically found in robotic scenarios.

### 2.1 The Dynamics of Neural Fields

**Figure 1:**
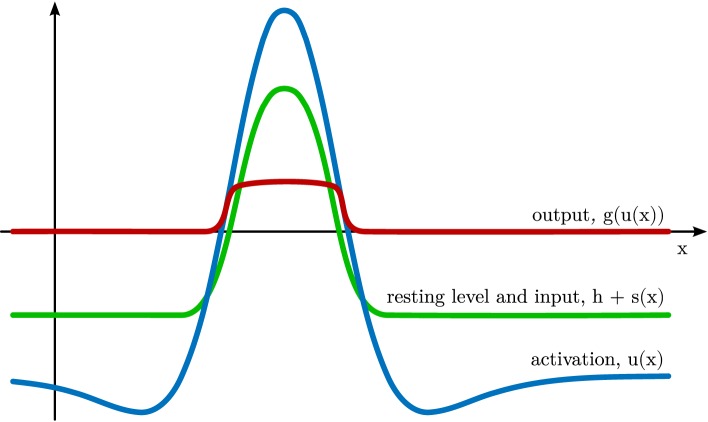
**Dynamic neural field defined over a one-dimensional feature space *x***. The activation *u*(*x*) of the field is plotted in blue, the subthreshold attractor *h* + *s*(*x*) in green, and the output *g*(*u*(*x*)) in red.

*Dynamic neural fields* are the core elements of DFT. A field consists of a distribution of activation, *u*(**x**,*t*), defined over one or more continuous metric feature dimensions **x** = (*x*_1_, …, *x_n_*) (see Figure 1 for a one-dimensional example). The activation of a field evolves in time, *t*, according to the neural dynamics

(1) $$\tau \dot{u}\left(x,t\right)=-u\left(x,t\right)+h+s\left(x,t\right)+c_{\text{noise}}\;\xi \left(x,t\right)+\int \cdots \int k\left(x-x\prime \right)\;g\left(u\left(x\prime ,t\right)\right)dx\prime .$$

**Figure 2:**
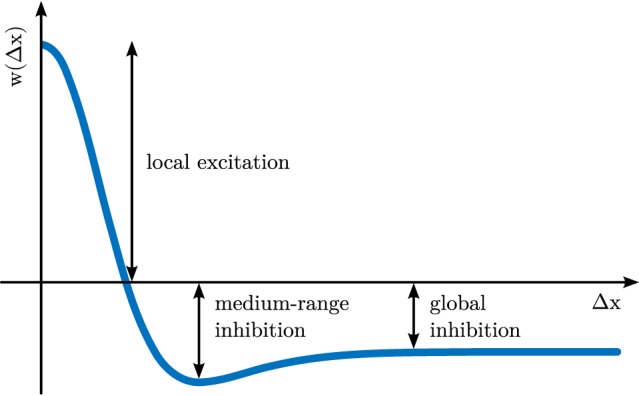
**Interaction kernel defined over distance, Δ*x*, within a one-dimensional feature space, *x***.

The parameter *τ* defines the time scale of the dynamics. It scales the rate of change of activation, $\dot{u}\left(x,t\right)$, which is inversely proportional to the current level of activation at the same location, −*u*(**x**, *t*). On its own, the −*u*-term creates an attractor at *u*(**x**) = 0 and is thus ultimately responsible for creating the field’s stability properties. The negative constant, *h* < 0, is the resting level of the dynamics. It shifts the attractor, so that the activation relaxes to *u*(**x**) = *h* in the absence of any other inputs. Location-dependent input, *s*(**x**, *t*) may shift this attractor upwards. At each field location, Gaussian white noise, ξ(**x**, *t*), adds random perturbations to the field, scaled with a noise strength, *c*_noise_. Finally, the integral term describes neural interaction, which is positive for neighboring locations (*local excitation*) and negative for all or distant locations (*global inhibition*), as characterized by the interaction kernel, *k*(Δ**x**) (see Figure 2 for a one-dimensional example).

Interaction only comes into effect when activation is above a threshold, as characterized by a sigmoidal output function, *g*(*u*(**x**)) ∈ [0, 1]. Different variants of the output functions may be used. In Amari (1977), the output function is a step function. Another common choice is the logistic function. We often use a computationally more efficient approximation of the logistic function given by

(2) $$g\left(u\right)=\frac{1}{2}\left(1+\frac{\mathrm{\beta u}}{1+\beta |u|}\right).$$

Given sufficient external input that is localized in a region along the feature dimensions, the field creates a localized *peak* of suprathreshold activation as an attractor [see Figure 1 for a sketch; for an in-depth analysis, see Amari (1977); Taylor (1999)].

In dynamic field theory, such stable peaks of activation are the units of representation. The position of a peak along the metric dimensions, **x**, determines which metric values it represents.

Different configurations of stable solutions emerge depending on the strength and spatial structure of inputs as well as on parameter values in the interaction kernel. Changes in any of these may lead to transitions from one set of stable states to another. These transitions are dynamic *instabilities* from which basic cognitive functions emerge. The *detection instability* occurs if excitatory input pushes a subthreshold activation pattern above threshold so that local excitatory interaction starts taking effect. This further raises activation around the localized input. Activation thus diverges from the subthreshold pattern and converges to a localized peak of activation. Only if the input level drops significantly below the level at which the initial detection instability occurred does a *reverse detection instability* induce the decay of the localized peak to subthreshold levels.

The level of input at which a reverse detection instability occurs depends on the strength of excitatory interaction. For sufficiently strong excitatory interaction, the reverse detection instability may no longer occur for any (non-negative) input value. In this case, a peak remains stable even when the inducing localized input has been removed. The peak location reflects past localized inputs, a model of working memory referred to as *sustained activation*. The parameter configuration at which this solution emerges is the *working memory instability*.

When interaction is mainly local, multiple peaks may be present at the same time. Global inhibition may lead to selection, in which a single peak suppresses all other localized inputs. Such a selection decision may be multi-stable, in the sense that any of the multiple peak locations may become realized depending on the history of activation and stimulation. When input strengths are sufficiently different at different field locations, this multi-stability may break down in the *selection instability*, in which a unique “winner” of the implied competition emerges.

### 2.2 The Dynamics of Neural Nodes

Dynamic neural nodes (or nodes for short) are zero-dimensional neural fields

(3) $$\tau \dot{u}\left(t\right)=-u\left(t\right)+h+c_{\mathrm{uu}}\;g\left(u\left(t\right)\right)+s\left(t\right)+c_{\text{noise}}\;\xi \left(t\right),$$

in which the feature dimension consists of a single point represented by the activation variable itself. The only interaction is then self-excitation of strength *c_uu_* > 0. Dynamic neural nodes may undergo detection and reverse detection instabilities and may also form working memory, that is, remain activated after *s*(*t*) has returned to zero. Selectivity is not meaningful for a single node. Multiple nodes may form competitive networks through mutual inhibitory coupling (see below). Such networks may perform selection and undergo a selection instability.

Nodes are used in DFT architectures to represent categorical states. Although these could be envisioned to be embedded within continuous spaces, the lack of an obvious topology may make it useful to represent them by isolated nodes. Prominent examples of categorical states are ordinal nodes that represent where in a serially ordered sequence of events a particular behavior or representation is activated (Sandamirskaya et al., 2011). Relatedly, different behaviors may be activated or deactivated by dynamic neural nodes in what we call *behavioral organization* (Richter et al., 2012). Categorical concepts may likewise be represented by nodes (Richter et al., 2014b).

### 2.3 The Dynamics of Memory Traces

Dynamic memory traces account for synaptic changes and long-term memory effects, such as habit formation (Schöner and Dineva, 2007) and habituation (Schöner and Thelen, 2006). A memory trace is modeled as a distribution of activation over a specific feature space, much like a dynamic neural field. However, the dynamics governing the memory trace differs from the neural dynamics of fields. It receives input from the output of an associated dynamic neural field and is essentially a low-pass filter of that output, evolving on a slower time scale. A number of different mathematical formulations are used [see Sandamirskaya (2014) for review]. Memory traces generate distributions of activation that reflect the history of activation in the associated field. They may estimate the probability distribution of peak events in that field (Erlhagen and Schöner, 2002). Memory traces are typically fed back into the associated field, which they preshape, favoring the generation of the previously activated patterns.

### 2.4 The Coupling of Neural Fields and Nodes

Multiple fields, nodes, and memory traces may be coupled to build DFT architectures (Zibner et al., 2011a; Zibner and Faubel, 2015). We explain the different kinds of coupling functions in reference to a source field, *A*, of dimensionality *a* and a target field, *B*, of dimensionality *b*. Consider first the simplest case, *one-to-one coupling*, in which the two fields have the same dimensionality (*b* = *a*). It is always implied that the dimensions of the two fields are aligned with each other, so that the source field, *u_A_*(**x**, *t*), is defined over the same vector, **x**, as the target field, *u_B_*(**x**, *t*). The software framework *cedar* (see Section 4) provides routines for remapping entries of the vectors when the dimensions of *A* and *B* are not correctly aligned.

Coupling means that the output of the source field, *g*(*u_A_*(**x**, *t*)), is an additive contribution, *s_B,A_*(**x**), to the external input, *s_B_*(**x**, *t*), of the target field *B*. For one-to-one coupling,

(4) $$s_{B,A}\left(x,t\right)=g\left(u\left(x,t\right)\right).$$

When the target field *B* represents more metric dimensions than the source field *A* (*b* > *a*), the coupling is an *expansion*. This means that the vector, **x***_B_*, which describes the dimensions of the target field contains all dimensions of the source field, **x***_A_*, but has additional entries not contained in the source field. In expansion coupling,

(5) $$s_{B,A}\left(x_{B},t\right)=g\left(u\left(x_{A},t\right)\right),$$

the right hand side does not depend on these extra dimensions of **x***_B_*. Input is, therefore, constant along those additional dimensions [ridge or tube input in two or three dimensions; see Zibner et al. (2011a)]. In *cedar*, functions can be used to arrange which slots of **x***_B_* receive constant input.

When the target field *B* represents fewer metric dimensions than the source field *A* (*b* < *a*), the coupling is a *contraction*. Some dimensions, on which the source field *A* depends, are not represented in the target field *B*. We assume these extra dimensions of the source field are the last (*a* − *b*) slots, *x_b_*_+1_, …, *x_a_*, of **x***_A_* (again, *cedar* functions can be used to arrange that). There are multiple possible ways how the dependence of activation on these extra dimensions may be contracted. The most common form is to take an integral over the extra dimensions:

(6) $$s_{B,A}\left(x_{B}\right)=\int \cdots \int g\left(u_{A}\left(x_{A}\right)\right)\;dx_{b+1},\ldots ,{\mathrm{dx}}_{a}.$$

Couplings between fields and nodes are covered by these same principles. For example, the expansion from a node *A* to a one-dimensional field *B* provides input to the field,

(7) $$s_{B,A}\left(x,t\right)=g\left(u\left(t\right)\right).$$

This implements a global *boost* to the target field, a mechanism often used to induce detection instabilities.

The contraction from a one-dimensional field *A* to a node *B*,

(8) $$s_{B,A}\left(t\right)=\int g\left(u\left(x,t\right)\right)\mathrm{dx},$$

may implement a *peak detector*: under appropriate choice of model parameters, any peak occurring in the source field may push the node through the detection instability.

Input from couplings, s*_B,A_*(**x**, *t*), may be further transformed before being added to the neural dynamics of the target field. A common form is to apply a weighting function, *c*(**x***_B_*):

(9) $$\tau {\dot{u}}_{B}\left(x_{B},t\right)=\cdots +c\left(x_{B}\right)\;s_{B,A}\left(x_{B},t\right).$$

Another common transformation is to convolve the input with a kernel, *k* (Zibner et al., 2011a):

(10) $$\tau {\dot{u}}_{B}\left(x_{B},t\right)=\cdots +\int \cdots \int k\left(x_{B}-x_{B}^{\prime }\right)\;s_{B,A}\left(x_{B}^{\prime },t\right)\;{dx}_{B}^{\prime }.$$

The kernel is often chosen as a Gaussian that spreads input to neighboring sites that represent similar feature values.

### 2.5 The Coupling of Neural Fields to Sensors and Effectors

DFT provides concepts for how to integrate sensory information into cognitive architectures and for how to drive effector systems based on the neural representations generated within a cognitive architecture.

#### 2.5.1 Sensors

Sensors provide input to DFT architectures. Mathematically, this means that sensory data determine the values of input functions, *s*(**x**, *t*), to relevant fields of the architecture. These functions are defined over relevant feature dimensions, **x**. Sensory data may be represented in these input functions in two ways. They may set the amplitude of the input function, *s*. This is neurally interpreted as a form of rate code, in which different levels of activation stand for different sensory events. On the other hand, feature values obtained from sensory data may be represented within the feature dimension, **x**. Neurally, this corresponds to space or population code.

For example, a color camera may deliver hue and saturation values for each pixel. The input function, *s*(**x**, *t*), derived from such a camera may be defined over the feature space **x** = (*x, y, h*), where *x* and *y* are Cartesian coordinates in the camera plane, and *h* is hue. For every location, (*x, y*), only the point along the hue axis that represents the hue value currently returned by the camera at the corresponding pixel generates non-zero input. Everywhere else along the hue axis, the input function is zero. The amplitude of the entry at the matching hue value is the saturation reported by the camera at that pixel. Formally:

(11) $$s\left(x,t\right)=\left\{\begin{array}{cc} S\; & \text{for}\;h=\text{hue value returned at pixel}\left(x,y\right)\text{at time}\;t\\ 0\; & \text{otherwise}, \end{array}\right.$$

where *S* is the saturation level returned by the camera at pixel (*x, y*) at time *t*. Space codes may distribute input values more smoothly along the feature axis, for instance, by applying a Gaussian filter along the feature dimension. Input distributions over feature spaces may also be derived from preprocessing operations applied to the raw sensory data. For example, batteries of edge filters may generate different levels of input at different spatial orientations for each location in a visual input.

#### 2.5.2 Effectors

Ultimately, a neural field may be used to control an effector by specifying a motor command. Typically, such a command is a specific value, say a vector **x**_cmd_, which is contained within the dimension, **x**, over which the field, *u*(**x**, *t*), is defined. Specifying the motor command thus amounts to “reading out” a value from the neural field. Intuitively, the location of maximal activation would seem the best choice for such a read out. In neural networks, this intuition is sometimes realized by a “winner takes all” mechanism. Such a mechanism is implemented in DFT by the competitive selection of a single localized peak of activation (which also ensures the stability of the selection decision). The problem that then remains is to extract the location of the peak along the dimensions of the field.

This seemingly trivial step runs into a problem of normalization (Kopecz and Schöner, 1995; Zibner et al., 2011a; Schöner et al., 2015a). A common idea is that the activation peak, passed through a sigmoid threshold function, *g*(*u*(**x**, *t*)), is used as a probability density over the field dimension, x, so that the expected value (or theoretical mean) of the field dimension is the estimate of the peak location:

(12) $$x_{\text{cmd}}\left(t\right)=\frac{1}{N}\int x\;g\left(u\left(x,t\right)\right)\;dx.$$

This is only an unbiased estimator of the peak location if the probability density is correctly normalized by

(13) N=∫g(u(x,t))dx.

The obvious problem arises when no peak is generated and *N* = 0.

This normalization problem disappears when motor control is also thought of in dynamical systems terms. The problem is then no longer to compute **x**_cmd_(*t*), but to create a dynamical system of a control variable, **x**_ctrl_, which has an attractor at **x**_ctrl_ = **x**_cmd_(*t*) that may vary in time slowly enough for the control dynamics to track the change. This can be achieved without normalization by realizing that the attractor should become unstable when the peak disappears:

(14) $$\tau_{\text{ctrl}}\;{\dot{x}}_{\text{ctrl}}=-N\left(x_{\text{ctrl}}-x_{\text{cmd}}\right)$$

where *τ*_ctrl_ is a time scale. This dynamics has an attractor for **x**_ctrl_ at **x**_cmd_, which becomes marginally stable when *N* goes to zero. That removes the problem of normalization. To see this, resolve the parenthesis on the right hand side, insert equations (12) and (13) for **x**_cmd_ and *N*, and rearrange the terms under a single integral:

(15) $$\tau_{\text{ctrl}}\;{\dot{x}}_{\text{ctrl}}=-\int \left(x_{\text{ctrl}}-x\right)g\left(u\left(x,t\right)\right)\;dx$$

This formulation no longer requires the direct estimate of **x**_cmd_.

## 3 Implementing Dynamic Field Theory

DFT architectures are typically solved numerically on a digital computer. This may serve to simulate DFT models based on artificial inputs that emulate experimental paradigms. This may also serve to implement DFT models in artificial cognitive systems, such as autonomous robots or artificial perception systems. In such cases, the numerical solution of the neural dynamics must respect real-time constraints as current sensory readings are fed directly into the DFT architecture, which may drive effectors. We step through the issues that must be addressed in such numerical solution of the neural dynamics with respect to the sampling time, synchronization, the sampling of space, and the order in which coupled subsystems of DFT architectures are updated.

### 3.1 Sampling Time

The forward Euler method is the simplest algorithm for solving differential equations, although it has the lowest order of convergence. Even so, it is the method we chose to realize in implementation for a variety of reasons. First, the rate of the numerical approximation of the neural dynamics is limited by the rate at which sensor readings can be obtained. Methods of higher order such as Runge–Kutta require multiple evaluations at intermediate time steps. This implies that sensory channels are sampled at a higher rate than the motor output is generated. This is a complication and limits the advantage gained by higher orders. Methods with adaptive step size are not suitable when the evolution in time of sensory readings must be monitored. Moreover, the neural dynamics that governs a typical neural field [see equation (1)] is a stochastic differential equation. Higher order numerical methods for stochastic differential equations require very many function evaluations per time step (Kloeden and Platen, 1999), which defeats their computational advantage when each evaluation is computationally costly. Using the low-order Euler method is not a problem in DFT because the functional states of DFT architectures are attractors. Their stability properties also help stabilize the numerical approximation of the underlying differential equations, reducing the demands on numerical precision and enabling larger steps sizes compared to generic differential equations.

For a stochastic differential equation

(16) $$\tau \dot{u}=f\left(u\right)+c_{\text{noise}}\;\xi \left(t\right)$$

with deterministic dynamics, *f* (*u*), and Gaussian white noise of unit variance, ξ(*t*), the stochastic Euler method is

(17) $$u\left(t_{i}\right)=u\left(t_{i-1}\right)+\frac{1}{\tau }\left(\Delta t_{i}\;f\left(u\left(t_{i-1}\right)\right)+\sqrt{\Delta t_{i}}\;c_{\text{noise}\;}\xi_{i-1}\right).$$

Here, *t_i_* is a discrete sampling of time (*i* = 1, 2, …), which is approximately (but not strictly, see below) equidistant, Δ*t_i_* = *t_i_ − t_i − _*_1_, and ξ*_i − _*_1_ is the return of a Gaussian pseudo-random number generator. *u*(*t_i_*) is then a discrete time approximation of *u*(*t*). Note how the stochastic term scales only with the square root of the time step, while the deterministic term is linear in the time step [see, e.g., Zwillinger (1989), p. 584].

The time step, Δ*t_i_*, must be chosen such that the Euler approach provides a good numerical estimate of the underlying dynamics. To minimize computational effort, the largest possible time step is desired. How large the chosen value for the time step can be chosen depends on the time scale of the simulated dynamics. Theoretically, the time step needs to be several orders of magnitude smaller than the shortest time scale of the dynamics. In practice, the fact that attractor solutions help stabilize the numerical procedure means that we can use relatively crude sampling without running into numerical instabilities. We have often used time steps that were only one order of magnitude smaller than the relaxation time of the dynamics.

### 3.2 Synchronizing Real and Simulated Time

When DFT architectures are simulated off-line based on simulated inputs, the real physical time a computer program takes to update the dynamical variables matters only with respect to how long we must wait for the simulation to finish. However, when DFT architectures are used in artificial cognitive systems that are tied to real sensory data and drive autonomous robots, the alignment of the physical time, when the computer provides a new value for the dynamical variables, with the simulated time, *t_i_*, is important. In this case, which we now examine, another kind of constraint arises for the choice of the time step.

Clearly, if the computer systematically takes longer to provide an update of the dynamic variables than the Euler time step, then the dynamics cannot be realized on the artificial cognitive system. The real-time step may then be too long for the discrete time series to be a good approximation of the dynamics. As a result, the response of the robot or artificial perceptual system to time-varying inputs can no longer be predicted from the dynamics. The Euler time step has to be increased, so that the computer manages to provide the update within a time interval that is smaller than the chosen Euler time step.

This reverses the direction in which choices of parameter values are made: the computation cycle determines the fastest possible Euler time step, and that time step in turn determines how fast the dynamics may be. In other words, the time scales of the dynamics must be adjusted such that the dynamics can be consistently approximated in real time. If the computational cycle is the limiting factor, then the price to be paid is that the system has a limitation to how fast changes in its sensory inputs may be. Only changes that are slower than the slowest time scale of the neural dynamics can be tracked by the neural activation states.

**Figure 3:**
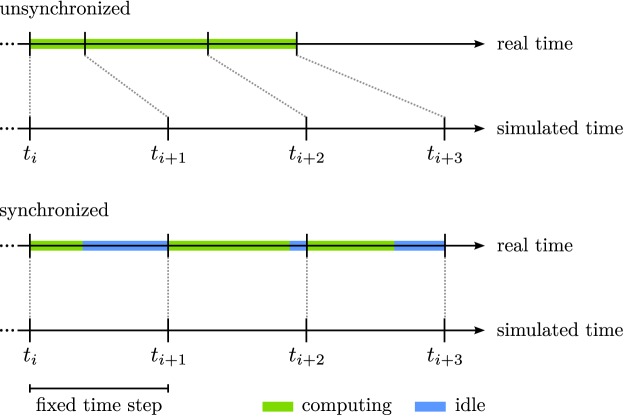
**The synchronization of real and simulated time**. The top panel illustrates that computing updates (green bars) as fast as possible may cause real and simulated time to become asynchronous. The bottom panel illustrates that waiting between updates (blue bars) until the end of the fixed time step addresses this issue.

Ideally, computation time is not a concern so that the computer is fast enough to provide updates within the time interval that is an adequate time step for the dynamics with the desired time scales. Even in such a case, care must be taken that the physical time, at which the updates of the dynamical variables are provided by the computer program, does not become systematically desynchronized with the simulated time of the differential equation. This is illustrated in Figure 3 (top panel). Were we to start a new evaluation of the numerical solution every time the computer program has provided an update, then the time of the dynamics, captured by *t_i_*, would become increasingly out of tune with the physical time (in a sense, the time of the dynamics would run ahead of physical time). Again, the properties of the neural dynamics would no longer be inherited by the physical implementation.

The simple solution is to wait with the initiation of a new computational step until the desired Euler step has passed (bottom panel of Figure 3). This is implemented in *cedar* as a mode of clocking the computational update cycle. The *cedar* implementation also addresses the opposite limit case in which a computational cycle occasionally takes longer than the desired Euler step. In this case, *cedar* extends the Euler step, Δ*t_i_*, at the next update to bring the time of the dynamics back in line with physical time. Clearly, this must happen only occasionally, lest the Euler step becomes consistently longer than planned and the goodness of numerical fit suffers. *cedar* provides a “meter” which indicates how often such adaptive changes to the Euler step take place. When too many events of this kind occur, the meter prompts the user to reparameterize the dynamics and lengthen the planned Euler step.

A computer that implements a DFT architecture of an artificial cognitive system will typically not operate in strictly deterministic time as many processes, some unrelated to the implementation, share processor time. As a result, the actual computational cycle will fluctuate. A positive side effect of waiting to the desired Euler step is that such fluctuations are minimized. Only instances in which the cycle is longer than desired leave a trace in the time of the dynamical system.

DFT architectures can become large and complex, potentially including dozens of fields of different dimensionality (Zibner et al., 2011b; Richter et al., 2012; Knips et al., 2014). This may ultimately pose challenges to the real-time updating of the solutions by the numerics. One possibility to optimize computational effort is to recognize that not all component fields may require the same Euler time step. For instance, some fields are not exposed to input that varies at the same rate as others. Higher dimensional fields impose disproportionately larger computational cost, so running such fields at lower rates is attractive.

A practical way to implement different Euler time steps is to carve up an architecture into components that are computed in separate threads of execution. This has the added advantage that the computation can be performed in parallel on multi-core CPUs. *cedar* provides this option when DFT architectures are designed. This approach implies that slight asynchronies may arise when the threads interact. Each thread reads output of other threads at times that may come from time samples that deviate from its own current time step. Our approach to the sampling of time guarantees, however, that the discrete time steps remain close to physical time. So these asynchronies do not accumulate and are thus small, of the order of one time step (the largest time step in the worst case). This is not a problem in practice, therefore, as we have observed empirically as well. This approach is also useful in accommodating constraints on cycle times that come from sensor or effector hardware.

### 3.3 Sampling Space

Activation fields are defined over continuous dimensions, which need to be discretely sampled for numerical evaluation by grids of the appropriate dimensionality. A simple rectangle rule is used to transform the integrals into sums. Integrodifferential equations are particularly well-behaved under discretization as they effectively filter discretization error, so this simplest approach works reliably. When fields are coupled that are defined over different size grids, the output of one field must be resampled to determine the input to the other field. Different interpolation methods for such resampling are available in *cedar*.^2^

The grid sampling deals correctly with the convolutions (with an odd number of sample steps) to provide unbiased estimates. The convolutions require padding of the fields. The default is padding with periodic boundary conditions, although other options are available for one- and two-dimensional fields. Convolution kernels are decomposed into separable components so that convolutions can be done separately along each dimension. Convolutions in three and more dimensions exploit fast Fourier transform (FFT) for computational efficacy. In two dimensions, FFT is used depending on the grid size.

## 4 Architectures in Dynamic Field Theory

To provide neural process models of cognitive function within the framework of dynamic field theory, typically entire architectures must be built. We outline the issues that must be addressed when DFT architectures are built and introduce elements of *cedar* that help solve these problems.

In the information processing paradigm, cognitive architectures are designed in terms of modules that can be characterized by input/output functions. While the architecture organizes the flow of information, the actual processing is done by the individual modules that realize a particular function. When such architectures are used to build artificial cognitive systems, not only must the architecture be specified but also the individual functions must be programmed to deliver the respective functionality. These functions are, by themselves, relatively unconstrained.

In DFT, in contrast, all components of an architecture are either dynamic fields or dynamic nodes, whose function is constrained by the same differential equation throughout the architecture. The only extent to which the function of each component can be adjusted is by “tuning” its parameters to determine one of a limited number of dynamic regimes. For example, fields may be in the mode in which only one self-stabilized peak may be induced at a time. They may also be in a multi-peak regime, in which the peaks may be sustained or may depend on localized input.

What the activation within each dynamic field or node represents is determined by how the field or node is connected to the rest of the architecture, and ultimately to the sensory and motor surfaces. The ways in which fields and nodes are coupled are also highly constrained, as we outlined earlier. Activation patterns output by one field may provide excitatory or inhibitory input to another field or node. Which outputs may be available as input to any given field or node is determined by the architecture.

Applying an “operator” (e.g., adding) to two inputs, for instance, is achieved by a coupling structure, in which every location in both input fields is connected to any possible location in the target field. This amounts to a coordinate transform (Schneegans and Schöner, 2012). Once implemented within DFT, an operator can become part of a stable coupling from time-varying sensory inputs to motor control.

**Figure 4:**
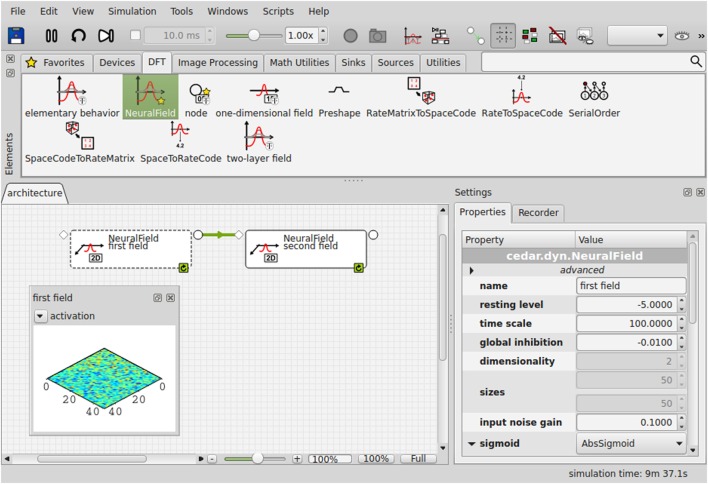
**The graphical user interface of *cedar***.

Building a DFT architecture that realizes a particular cognitive function thus amounts to specifying the dynamic elements, fields and nodes, their dynamic regimes, and the coupling structure. The constraints in all three aspects make it both possible and attractive to provide a software framework within which DFT architectures can be built. *cedar* (Lomp et al., 2013) is such a software framework. In *cedar*’s graphical user interface (Figure 4), the different components of DFT architectures are available as icons in an element panel. Dragging the icons into the architecture canvas instantiates the corresponding field or node. The field and node represented by icons can be coupled by graphically connecting the output slots on one icon to the input slots on another icon. Contraction or expansion of field dimensions can be specified for each connection.

*cedar* thus makes it easy to specify DFT architectures that can reach considerable complexity (a simple example be elaborated in the next section). The result is one big dynamical system, automatically instantiated by *cedar*. The remaining task is to “tune” the system by choosing values for the parameters of the different neural dynamics that include resting level, input strength, and the strength and spatial range of excitatory and inhibitory interaction. The mathematical framework of DFT imposes homogeneity within each field, effectively reducing the potentially high-dimensional set of neural connections to a small number of kernel parameters. Similarly, the constraints on coupling functions within DFT leads to a reduced set of coupling parameters that does not depend on how the fields are sampled along their dimension (in contrast, for instance, to how the number of connection weights grows with the number of neurons in connectionist networks).

The difficulty of tuning of DFT architectures is further reduced by an approximate form of modularity. The dynamic mode in which each field or node operates determines its function. That mode and thus function remains invariant as the dynamics of other components of the architecture vary until an instability is reached. Tuning thus amounts to ensuring that such instabilities occur only when and where desired. As a result, the onerous task of tuning analog computers, in which any change in any component may affect any other component, is much reduced in DFT. This also means that the “diagnostic” for correct function is local and often qualitative. The goal is to ensure that the right kind and number of peaks are formed under the right circumstances in each field. Assessing the performance of a DFT architecture does not always require an estimate of the continuous activation patterns along all dimensions and fields at the same time.

What remains a challenge to tuning the parameters of DFT architectures is to scan the range of relevant inputs. Simulations of experimental paradigms rely on the same restrictions psychophysicists impose on humans to limit the range of stimuli used. But building artificial cognitive systems requires that a range of potentially naturalistic inputs must generate the desired behavior and cognition. Tuning DFT architectures while they are linked to naturalistic inputs from real sensors is challenging but made easier in *cedar*. The task then remains to vary the physical stimulation provided and assess the state of the DFT architecture. *cedar* supports this task by providing flexible visualization of the dynamic state of any element, delivered in real time (illustrated in Figure 4). Such visualization can be used to assess the qualitative state of any field (e.g., number and identity of peaks). Quantitative assessment may make use of *cedar*’s recording functions (see below).

## 5 Workflow to Develop and Evaluate DFT Architectures

Developing and evaluating DFT architectures involves a sequence of steps: (1) conceiving of an architecture, (2) building it to enable simulation or implementation on an artificial cognitive agent, (3) tuning its parameters, (4) evaluating its performance, and (5) documenting the system. Often these steps must be iterated as an architecture is expanded or updated. The resulting workflow is outlined in this section around a simple, but exemplary problem. Our emphasis is on artificial cognitive systems that may be realized as autonomous robots or perception devices. We will use *cedar* to make each step concrete. DFT architectures may also be used to account for experimental data obtained in specific experimental paradigms. We refer to that problem only briefly here and point the reader to Ambrose et al. (2015) for an extensive review of the workflow in that context (that review refers to the MATLAB-based framework COSIVINA rather than to *cedar*).

**Figure 5:**
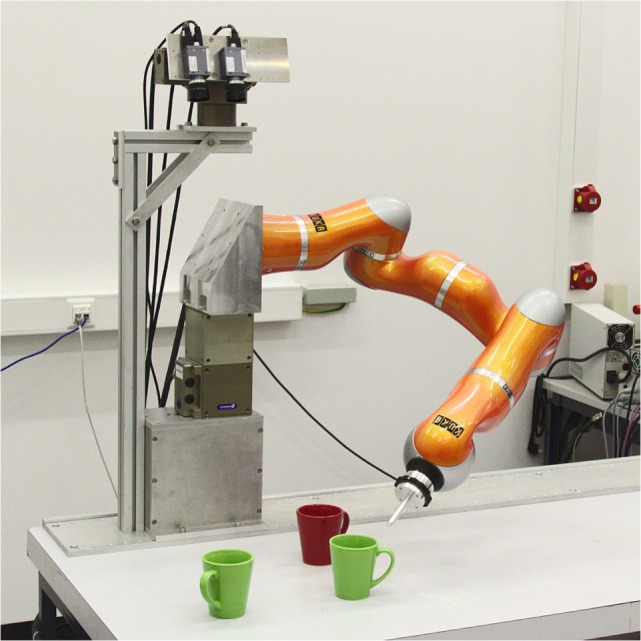
**The robotic setup for our object-oriented action task**.

**Figure 6:**
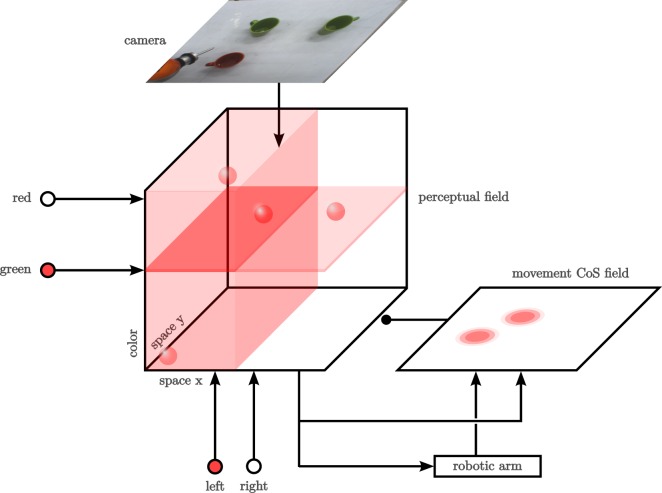
**Exemplary DFT architecture that does a simple feature search**. Activation levels in the architecture are schematically represented with shades of red, where more opaque colors represent higher levels of activation. Lines with normal arrow heads represent excitatory synaptic connections; lines ending in circles represent inhibitory connections.

The task we solve is a simple object-oriented action illustrated in Figure 5: an autonomous robot arm equipped with a camera examines an array of objects on a tabletop and points at the object in the scene that matches a description provided in terms of a feature cue (e.g., “green”) and a spatial term (e.g., “left”). The DFT architecture (Figure 6) is simple but makes use of both fields and nodes, couplings with both expansion and contraction, couplings to sensors and effectors, and many of the characteristic instabilities of the neural dynamics discussed earlier (Section 2). [For a more complete system of this general nature, see Bicho et al. (2010).]

### 5.1 Conceiving an Architecture

Developing a DFT architecture that solves the given task requires thinking about the sources of sensory specification, the means for action, and the cognitive properties implied by the task. Sensory specification is not only constrained by the sensors available (here a video camera) but also depends on the task (here, specification by color and space). Sensory specification leads to the identification of relevant dimensions of perceptual representations, here both space and hue value. Analogously, on the motor side, constraints that derive from the effector system together with the task lead to ideas about relevant motor dimensions (here, end-effector position in a Cartesian space). The requested cognitive properties may point to operators (here, kernels that ground spatial concepts “left” and “right”), to transformations (e.g., to achieve desired invariances; not a problem here, as the camera does not move), to problems of memory (e.g., to enable learning), or to problems of sequence generation (here, to first detect and then point).

The concepts of DFT are used to express the ideas derived from such an analysis. Feature or motor dimensions are represented in fields, concepts in nodes. Their coupling functions are used to realize operators and transformations. The dynamic regimes of the nodes and fields are used to create functions, such as detection, selection, and memory.

This translation of constraints and task demands into the language of DFT amounts to designing a DFT architecture, typically first sketched on paper, and “mentally simulating” it to specify the dynamic regimes and their interdependencies. Figure 6 is such a sketch for the present example, and we will step through this architecture now to illustrate the notion of “mental simulation.”

The perceptual representation on which this task can be realized is a three-dimensional *perceptual field* (center of Figure 6) defined over the tabletop (so in an allocentric reference frame) with hue as the third dimension. A colored, localized object is represented by a blob of activation (a peak in 3D), whose center indicates location and hue value of the object (there are four such blobs in the figure).

The camera provides a continuous stream of color images (top). These are in the camera frame, so they must be transformed to the allocentric coordinate frame. Each pixel delivers a hue value and its saturation. The pixel location transformed to the tabletop frame, and the hue value determines the 3D location in the field to which the input is directed. The saturation value determines the strength of the input.

The concepts used to cue the pointing target are represented by nodes. On the left, there are two nodes for “green” and “red.” At the bottom, there are two nodes for “left” and “right.” These nodes are in the bistable dynamic regime, so that they can be set by input from the user, reflecting the task specification. In the figure, the “green” and the “left” node have been set this way.

The coupling of the nodes into the perceptual field implements the relevant cognitive operation. For color, each node provides a sheet of input that is localized along the hue dimension but is homogeneous along the two spatial dimensions of the perceptual field. For the spatial terms, the “left” node projects onto the left half space homogeneously along the color dimension. The “right” node projects onto the right half space in the same way.

The idea is then that a single self-stabilized peak (blob) may arise in a detection instability when input from the camera is combined with input from the cue nodes. By operating the field in the single-peak dynamic regime, a single object is selected. This will be the object for which camera input overlaps best with cue input. In the figure, the green object in the top left wins the competition because it lies within both the “green” sheet and the “left” kernel. Without camera input, only subthreshold activation should be induced. Without both cues, localized camera input should likewise be insufficient to induce the detection instability. This form of “mental simulation” serves to identify the dynamic regimes the perception field must have under various conditions.

The output from the perceptual field goes directly into a system that controls the robot arm. The coupling function contracts along the color dimension, so that an activation pattern over the two spatial dimensions of the tabletop is handed to the robot arm. This activation pattern is transformed into an attractor dynamics for two variables that control the two Cartesian coordinates of the robot’s end-effector (the tip of its pointing tool). The vertical position of the attractor is fixed. This transformation from an activation field to an attractor dynamics is described in Section 2. Details are provided in Section S1 of the Data Sheet in the Supplementary Material.

The robot arm moves from its initial position to the attractor state. This takes time. The sequential organization of the task consists of initiating and terminating this movement. The movement is initiated when the peak in the perceptual field first arises in a detection instability. Termination of the movement is controlled by a *condition of satisfaction* (CoS) field defined over the two spatial dimensions of the tabletop and illustrated on the right of Figure 6. The movement CoS field receives input from the perceptual field reflecting the location of the selected target (inducing the rightmost subthreshold hill of activation). The CoS field also receives input from a simulated proprioceptive sensor that indicates the tabletop coordinates over which the tip of the robot’s pointer tool is positioned (inducing the leftmost subthreshold hill of activation). When the two sources of input overlap, the CoS field goes through a detection instability and generates a self-stabilized peak, which projects inhibitorily onto the perceptual field and all cue nodes. As a result, the perceptual field goes through a reverse detection instability, losing its peak, and the nodes switch into the deactivated state. The removal of input from the perceptual field makes the peak in the CoS field unstable. The CoS peak decays, and the CoS system returns to its initial state. So, reaching the selected target ends the movement and resets all fields and nodes to subthreshold values. The architecture is open to receiving a new cue. Here, “mental simulation” leads to a set of conditions under which instabilities in the different component fields and nodes must arise. These will be used to set the parameter values of the components as discussed below.

This form of specification of an architecture is limited in scope by the range of constraints that a designer can focus on at any given time. Architectures will typically be developed piece by piece. These pieces can be joined up due to the inherent (approximate) modularity of DFT architectures. Commonly, architectures are also developed in an incremental form, in which functioning portions may be expanded or updated to accommodate additional tasks or constraints, leading to an iterative specification process. Building and simulating the architecture is an important check on the validity of the “mental simulations.” The capacity to do this early in the specification process is a strength of *cedar* and a practical necessity in using the DFT framework for complex tasks.

### 5.2 Building DFT Architectures in *cedar*

Building a DFT architecture in a way that it can be solved numerically in simulation or on an artificial cognitive agent requires transforming the conceived model into computer code. In the past, one would have gone about that by first writing out all the mathematical equations that formalize the conceived model and then coding these equations in a computer program that solves the equations numerically. It is easy to visualize that the set of equations for even this relatively simple model is quite large. Notation for each dynamical variable or field and the associated parameters would need to be fixed. Updates during the iterative process of specifying the model would then often require rewriting such code.

**Figure 7:**
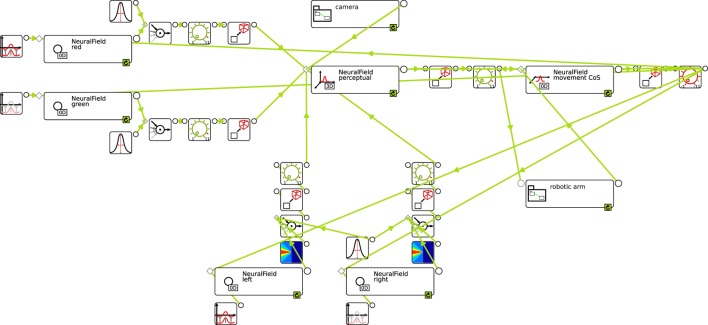
**The complete architecture assembled in *cedar***. The connection to the hardware level (sensor and motor) is hidden in the collapsible groups “camera” and “robotic arm.” The position of all elements on the canvas roughly resembles those in our earlier sketch of the architecture (Figure 6) to ease the comparison.

Modern software tools make it possible to shortcut this workflow by going directly from the conceived architecture, represented as a graphical sketch of the model, to its implementation in numerical software through a graphical programming interface. This is exactly the functionality that *cedar* provides. Figure 7 shows the end-result of such a graphical assembly process for the architecture we conceived in Figure 6.

In building DFT models in *cedar*, the coupling structures between dynamic elements typically consist of multiple processing steps, through which the range of possible couplings can be spanned. Let us look at one example of such a coupling structure, the projection from a color node (NeuralField red on top left) to the perceptual field (NeuralField perceptual in the top center). The color node is labeled as a NeuralField, but the instantiated field is zero-dimensional, so it is really just a single activation variable. Its output, the activation level passed through a sigmoid function, is multiplied in the first processing step (box with a circle and incoming arrows to the right of the color node) with a Gaussian kernel (box on top). The outcome is a one-dimensional vector that reflects the projection of the color node along the hue dimensions. This vector is multiplied in the second processing step with a constant, the strength of the coupling (box with a green dial). The third processing step is an expansion, in which the one-dimensional vector along the hue dimension is expanded homogeneously along the two spatial dimensions. This creates the sheet-like input pattern sketched in Figure 6. All other coupling structures contain similar processing steps, including in some instances, contractions and making use of other kernels (e.g., the somewhat triangular kernels for the spatial terms at the bottom).

The couplings from the camera (center top) and to the robot arm (bottom right) contain more complex series of processing steps that are detailed in Sections S1.1 and S1.2 of the Data Sheet in the Supplementary Material. These entail communicating with sensory and robotic hardware through the interprocess communication functions from the YARP library (Metta et al., 2006), which can also be used to run different parts of an architecture on different networked computers.

### 5.3 Tuning Parameters

As soon as the architecture has been graphically assembled in *cedar*, it can be numerically simulated. The parameter values of the architecture can thus be tuned by simulating the model using live inputs and observing the resultant activation patterns. The goal of tuning is to ensure that all neural fields and dynamic neural nodes are in the specified dynamic regimes. This also depends on the input patterns and their strengths, so being able to provide live input is critical. Some of the normalization problems when input varies are reduced by the sigmoid function and the relatively invariant shape of self-excited peaks of activation. Still, the remaining tuning task is often non-trivial so that tuning can be a demanding part of the workflow.

Tuning parameters online is facilitated in *cedar* by two features. First, parameter values can be changed (in the properties panel of the user interface) while the numerics is running, and these changes take immediate effect in the next update step of the Euler approximation. Second, the effects of such changes are instantly observable with *cedar*’s online plotting capabilities, through which the user can visualize the state of any element in a DFT architecture.

**Figure 8:**
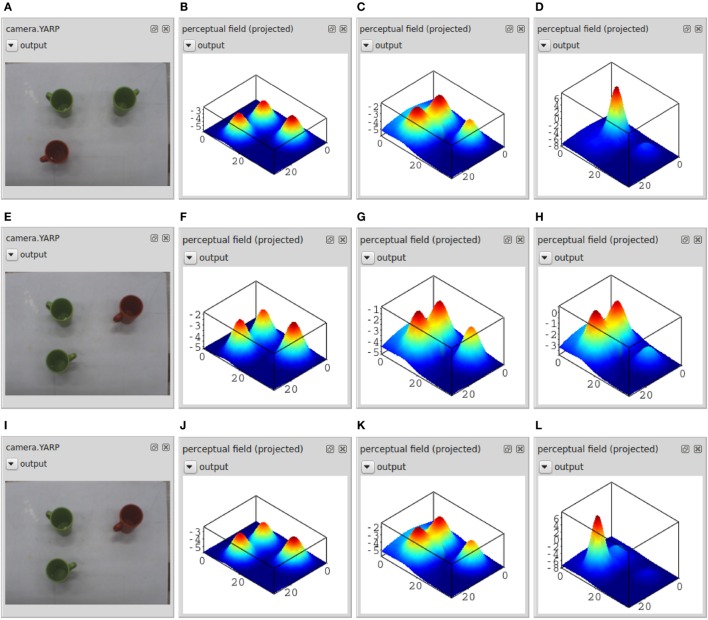
**For three different settings (three rows), this figure shows the camera input (leftmost column, A,E and I) and the resulting activation in the perceptual field at three different points in time (three rightmost columns): with none of the dynamic nodes activated (second column from the left, B,F and J), with the “left” node activated (third column, C,G and K), and with both the “left” and “green” node activated (fourth column, D,H and L)**. The three-dimensional activation of the perceptual field is shown here projected onto the two-dimensional table space.

Figure 8 illustrates online parameter tuning by showing the current camera input (left column) and the online plot of the perceptual field (summed along the hue dimension) at three different moments in time (rightmost three columns) in three different settings (three rows). On top, a scene was assembled in which a unique response should be obtained to the query “green” and “left” object. To tune the parameters of the perceptual field, the activation of the two nodes for “green” and “left” are controlled (through a user input panel). Parameters, here primarily the input strengths, are varied such that the field remains below threshold in the presence of camera input while both cue nodes (Figure 8B) or at least one cue node (Figure 8C) remain off. The system must go through the detection instability and generate a self-excited peak only once both cue nodes are activated and camera input contains an object matching the description (Figure 8D).

The middle and bottom row illustrate how the parameter values are refined to address selection. A scene is presented in which two objects match the cues “green” and “left.” In the middle row, two self-excited peaks are generated in the presence of both cues (right panel). The inhibitory coupling within the perceptual field is not strong enough to impose the single-peak dynamic mode specified for the field. By tuning the strength of inhibitory and excitatory components of its interaction kernel, the field can be brought into that mode as shown in the bottom row.

*cedar* offers additional features helpful for tuning such as the capacity to slow down or speed up time in the numerical solvers to enable the user to observe time courses conveniently. There are also tools to optimize performance, such as measuring computation time for individual components, which enables the user to find costly components and helps to make decisions about components that should be offloaded onto another CPU.

### 5.4 Running Experiments

The transition from the model to real-world operation of an artificial cognitive system can make use of intermediate steps, in which the activation time courses are used to drive *simulated robots* and/or in which sensory inputs come from prior *sensor recordings* rather than from closed loop live sensors. Running DFT architectures with simulated robots is useful to free oneself from the safety constraints and physical limitations of hardware in an early phase of testing. It also enables running large numbers of trials efficiently and to obtain statistical data from such tests. Using sensor recordings makes it possible to test architectures against reproducible input streams, useful, for instance, when different variants of an architecture are to be compared.

**Figure 9:**
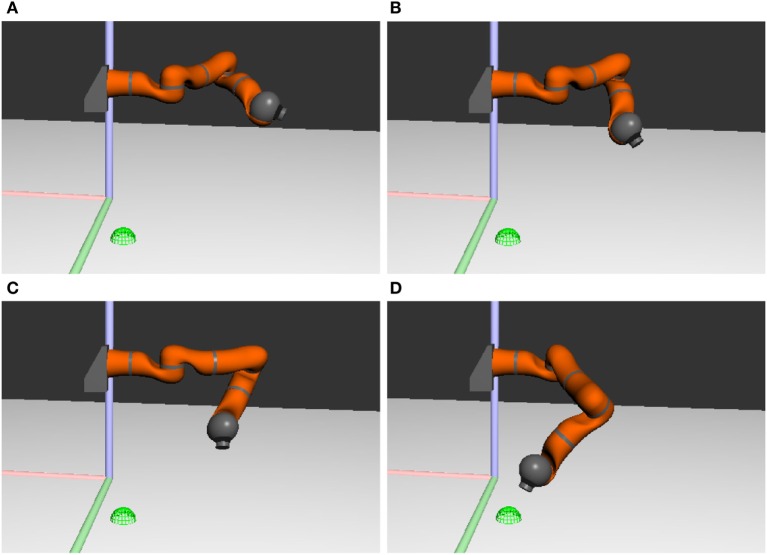
**Snapshots of the simulated robotic arm pointing toward a green mug on the left side of the table**. The target position is denoted by the green sphere. The colored lines show the coordinate axes of the world. Time increases from **(A)** to **(D)**.

*cedar* provides a built-in simulator of kinematic chains and color cameras overlooking a tabletop scene. This is illustrated in Figure 9 showing snapshots of the simulated robotic arm obtained at different moments of time from the architecture of Figure 7. For additional robots and more advanced simulation features (such as a full-fledged physics engine necessary for realistic object interactions), *cedar*’s network transparency can be used to interact with commercial robot simulator software [e.g., “Webots,” Michel (2004)]. *cedar* also features an *experimentation framework* that enables users to compile a set of conditions that trigger a list of actions associated with each condition. For the task of Figure 5, for instance, the experimentation framework could be used to automate the running of cued reaching experiments. At specific points in time (conditions), boosts may be specified to set the cue nodes (actions). Similarly, when the movement CoS field becomes active (condition), a trial could be terminated and a next trial started (action).

**Figure 10:**
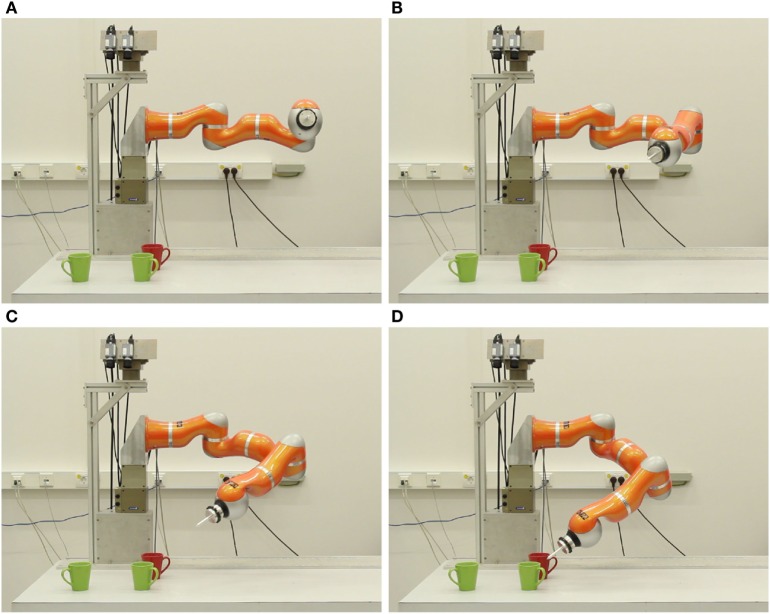
**Snapshots of the KUKA LWR4 robotic arm pointing toward a green mug on the left side of the table**. Time increases from **(A)** to **(D)**.

Ideally, the transition from simulated robotics to real-world robotics requires no or little additional parameter tuning. In *cedar*, this transition is brought about by replacing the simulated robot module by the corresponding real robot module. This directs the output to the hardware rather than the simulator. Figure 10 shows snapshots of the real robot arm acting out the same task as shown previously (Figure 9) in simulation.

### 5.5 Documenting Results

Characterizing the performance of an artificial cognitive system is not a trivial task. It requires defining some form of scenario or benchmark that probes relevant aspects of the desired cognitive function and the environmental conditions under which it is delivered. The time-continuous nature of processing in DFT architectures and their capacity to update processing online in response to changes in the environment make this task even more difficult.

**Figure 11:**
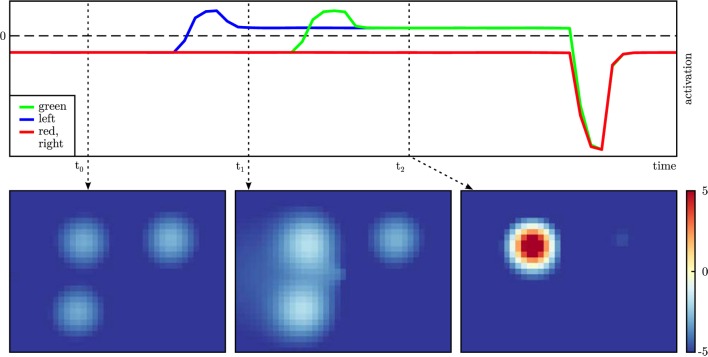
**Activation of the dynamic neural nodes of the exemplary architecture over time (top) with snapshots of the activation of the perceptual field at three relevant points in time (bottom)**. The activation of the nodes “red” and “right” have the same time course apart from noise. The perceptual field is projected onto the table space.

A key functionality of the *cedar* framework for evaluating DFT architectures is *data recording*, the capacity to register any data structure within an architecture (e.g., matrices of activation, sigmoid output, or projection stages) as a time series or as a snapshot. Time series are recorded at a user selected rate while the architecture is running and stored in CSV files (comma-separated values) together with a time stamp. Figure 11 illustrates how data obtained this way can be used to document real-world performance. The activation of the perceptual field and all dynamic neural nodes were recorded during an experiment performed with the real robot (Figure 10).

*cedar* also comes with a graphical tool that enables users to generate plots from recorded data. The tool is written in Python and uses the versatile matplotlib. Users can choose which data to plot and plotting modes of time course (e.g., activation over time), snapshot (i.e., the state of part of the architecture at a point in time), or a sequence of snapshots. High-dimensional fields can be projected onto lower dimensional spaces. The resulting plots can be saved as vector graphics (e.g., in SVG format) to include them in publications without loss of quality. This is how Figure 11 was produced.

## 6 Discussion

In this paper, we sketched the issues that must be addressed when neural cognitive architectures based on dynamic field theory (Schöner et al., 2015b) are developed to endow embodied agents with autonomy. The workflow of developing and evaluating such architectures was explained around a simple, but exemplary model system in which an artificial cognitive system points to an object within a visual scene that it selects based on a feature description (e.g., “the red object on the right”). Four stages of the workflow may be iterated, which are as follows: (1) The conception of the DFT architecture makes use of the concepts of DFT, specifying components as dynamic fields or nodes and their dynamic regimes in terms of the attractor states and their instabilities. “Mental simulation” of the conceived architecture uncovers the sequential logic in which instabilities must be induced in the model in response to external or internal signals. (2) As soon as a DFT architecture conceived as a graphical sketch is instantiated in the *cedar* framework through its graphical programming interface, the model can be simulated numerically. To do this, fields or nodes are dragged as icons from an element panel, and their coupling is specified by drawing connections within an architecture canvas. (3) Model parameters are tuned to realize the specified dynamic regimes. *cedar* provides online updating of parameter values with online visualization of any component of an architecture. (4) Assessing a DFT architecture in response to real or simulated sensory inputs is then possible by recording within *cedar* relevant inner states as well as the output of the architecture, for example, real or simulated robot motion. How *cedar* solves problems for users of the DFT framework is summarized in Table 1.

**Table 1:** **An overview of problems for users of dynamic field theory and *cedar*’s approach to solving them**.

Problem	*cedar*’s solution
Programming effort to implement DFT models	Graphical programming interface and object orientation minimize coding effort
Tuning parameters	Interactive inspection of model state and online updating of parameter values facilitate manual tuning of parameters; online slowing down of simulation time enables detailed inspection of dynamics
Handling large architectures	Graphical interface visualizes connectivity; *cedar* enables fast prototyping and incremental building of architectures
Embodiment	Built-in interfaces to hardware and simulation environments including “Webots” facilitate the design of autonomous robots
Collaboration	*cedar* architectures can easily be exchanged between users
Reproducibility	Virtual machines can be shared to enable any user to reproduce simulations without installing *cedar*
Dissemination of DFT framework	*cedar* can be used to implement DFT models without knowledge of programming languages
Sharing results	Simulation data may be recorded and shared for off-line analysis
Connecting to other frameworks	Plug-in infrastructure facilitates integrating functions from other frameworks; *cedar* supports YARP, and a ROS prototype is under development

This workflow is only feasible because DFT architectures can be built incrementally. This scaling property of DFT architectures ultimately comes from the stability constraint: in terms of dynamical systems theory, the function of a neural dynamics is captured by its solutions, the time courses of activation generated by the neural dynamics. To endow an individual component, a dynamic field, or node, with a particular function, we tune its parameters such that it has the desired dynamic regime as defined by attractor states. When other components are added to the model, the dynamic equation of the original component may change due to coupling. The solutions for the activation patterns of this particular component may then change as well. Because we generate functional states as attractors, the scaling requirement is merely that these attractors remain stable as new components are added. If that is the case, all solutions converging to the attractor are only changed in a graded way,^3^ which is sufficient to retain the function represented by the attractor. Attractors resist change not only in time but also when the dynamical equation is varied. As a result, the dynamic regime of any dynamic component typically remains invariant when the component is embedded in a larger architecture. This invariance is only approximate, so in practice, some retuning may be required.

Note that cognitive architectures based on classical models of information processing, such as ACT-R (Anderson, 1996) or SOAR (Laird et al., 1987), also have a systematic approach to scaling based on encapsulation. This comes, however, at the price of invoking mechanisms that are difficult to realize in neural process models, such as function calls and handing over arguments to operators. Neurally inspired approaches are beginning to overcome these limitations (Aisa et al., 2008; Jilk et al., 2008).

Beyond the toy example used here, DFT architectures have exploited the scaling properties of DFT to push both toward generating motor behaviors in autonomous robots (Knips et al., 2014; Strauss et al., 2015; Zibner et al., 2015) and toward higher cognitive function, such as grounding spatial language (Richter et al., 2014a), parsing action sequences (Lobato et al., 2015), or task learning (Sousa et al., 2015). These architectures are fairly complex. Designing them, tuning their parameters, and evaluating their performance was challenging. The workflow and its support by the *cedar* software framework presented in this paper were developed based on the experience of developing some of these models (which used preliminary versions of *cedar*).

The functionality of *cedar* may be extended beyond the theoretical language of dynamic field theory. User-supplied plug-ins may provide added functionality, such as other types of differential or integrodifferential equations, additional processing steps, new tools of visualization, and additional functions to improve online parameter tuning. *cedar* itself does not impose very constraining limits on the kind of functionality such extensions may provide. Still, the conceptual framework of *cedar* is particularly suited to continuous-time dynamical systems. Functionality that can be implemented through state variables that evolve in continuous time is thus integrable within *cedar* in the most direct way. Examples are the neural dynamics of the Hopfield type (Hopfield, 1999) and related continuous-time associative memories (Deco and Rolls, 2004) or the neural dynamics of central pattern generators (Ermentrout, 1998).

In a different context, neural models are aimed at modeling experimental data in particular behavioral paradigms. This context puts different demands on the conception, tuning, and evaluation of neural models. In particular, to simulate experimental paradigms, the task and set of sensory inputs must be captured and simulated, and measurements on the activation states of the models must be made that can be compared to behavioral observations. The workflow of modeling experimental paradigms within DFT was reviewed in Ambrose et al. (2015). A software framework, COSIVINA, written by Sebastian Schneegans in MATLAB, was specifically aimed at the development of DFT models that account for experimental data. COSIVINA facilitates scripting experimental paradigms and the collection and statistical analysis of simulation data. Unlike *cedar*, COSIVINA does not have a graphical programmer interface, and parameter tuning may become challenging once models become very large. The coupling to sensory and robotic hardware is central to *cedar*, but not, at this point, part of COSIVINA.

Other theoretical frameworks for neural models have developed analogous programming or simulation frameworks. Classical PDP models, for instance, can be efficiently assembled and simulated using pdp++ (O’Reilly and Munakata, 2000), now further developed and renamed “Emergent” (Aisa et al., 2008). Emergent has features that resemble both *cedar* and COSIVINA, having elements of a graphical programming interface, while also providing scripting that may be used to emulate experimental paradigms, which may be its main use case. Using the neural engineering framework of Eliasmith (2013) is facilitated by Nengo (Stewart et al., 2009), a software that also provides a graphical programming interface to specifying neural networks. Tuning parameters of neural architectures in robotic implementations that are situated in real environments is not a routine part of the workflow of these approaches.

A major motivation for the use of neurally inspired approaches in artificial cognitive systems is, of course, that they are open to learning. The approaches to learning provided by neural networks are well known. Typically, networks are learned up in training scenarios, in which stimulus patterns with or without supervisory information are used to update the connectivity of the network. Autonomously learning from experience is not as well understood. In robotics, reinforcement learning is used as a paradigm to learn from experience, but not typically within neurally grounded architectures [for review, see, Kormushev et al. (2013)]. DFT provides the processing infrastructure that supports autonomous learning from experience (Sandamirskaya, 2014; Sandamirskaya and Storck, 2015). For instance, neural states that drive exploratory behavior must be kept in working memory to compare with the outcome. Errors must be detected and represented, and the autonomous sequences of processing steps required to bring about an instance of experience must be generated. Even the simple, first examples of such autonomous learning within DFT required, therefore, relatively complex neural architectures, which were implemented using the *cedar* framework. While reviewing autonomous learning in greater depth is beyond the scope of this paper, it is a major frontier for future work.

## Author Contributions

OL, SZ, and MR developed *cedar*. MR implemented the architecture in the present paper and ran the experiments. All four authors contributed to the writing of the paper.

## Conflict of Interest Statement

The authors declare that the research was conducted in the absence of any commercial or financial relationships that could be construed as a potential conflict of interest. The reviewer RL declared a past collaboration with the author GS to the handling Editor, who ensured that the process met the standards of a fair and objective review.

## Footnotes

^1^*cedar* is open source C++ software licensed under the LGPL version 3. The source code, documentation, and other downloads can be accessed at http://cedar.ini.rub.de.

^2^In the current version of *cedar*, the grid step is one in each direction, so that it is not possible to vary the spatial resolution without a rescaling of the neural dynamics itself. This is merely an algorithmic simplification that could be improved upon if need be.

^3^The mathematical concept is that of structural stability (Arnold, 1983).
