# Supplementary material for: Developing Dynamic Field Theory Architectures for Embodied Cognitive Systems with cedar
Source: Front Neurorobot. 2016 Nov 2;10:14. doi: 10.3389/fnbot.2016.00014 (PMC5089998; doi:10.3389/fnbot.2016.00014)
Supplement: Supplementary file 1 [file Data_Sheet_1.PDF]

Supplementary Material:
Developing dynamic field theory architectures for embodied cognitive systems with *cedar*

Oliver Lomp *, **Mathis Richter, Stephan K. U. Zibner, and Gregor Schöner**
*Correspondence:
Oliver Lomp
oliver.lomp@ini.ruhr-uni-bochum.de

S1 CONNECTION TO THE SENSORY-MOTOR LEVEL

In this part of the supplementary material we take a more detailed look at the connection to the sensory-motor level, something we glossed over in describing our exemplary architecture (subsection 5.2 in the main article). First we show how we deal with the live camera input, transform it into table coordinates, and later into a representation that we feed directly into the perceptual field. Second, we show how we use the peak in the perceptual field to drive the robotic arm toward its target.

S1.1 Camera input and image preprocessing

When we implemented our exemplary architecture, we distributed the live system onto several computers that communicated over the network. We did this for two reasons: first, to demonstrate that it is possible in

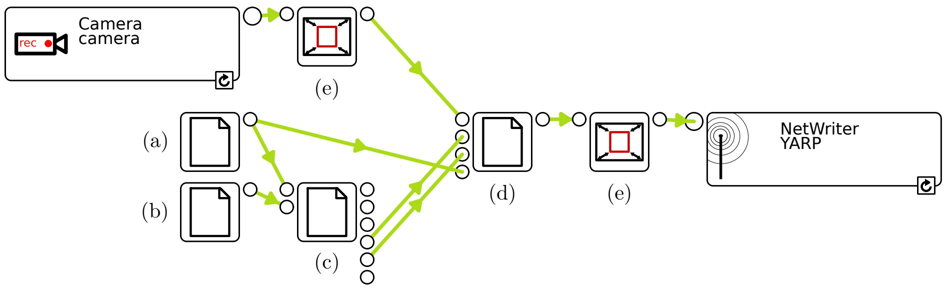

Figure S1. The part of the exemplary architecture (see Figure 7 in the main paper) that deals with grabbing the camera image and preprocessing it, before handing it over to another instance of cedar that is running on a different computer.

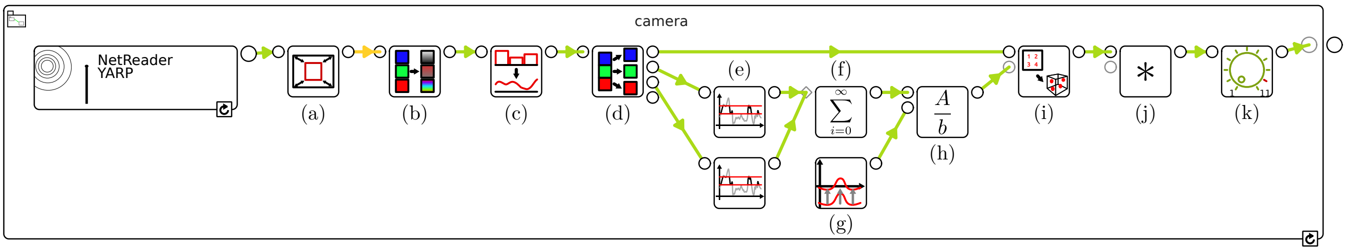

Figure S2. The part of the exemplary architecture (see Figure 7 in the main paper) that further preprocesses the camera image and feeds into the perceptual field. This figure shows the content of the group ‘camera’, which was shown in its collapsed state in Figure 7 in the main paper.

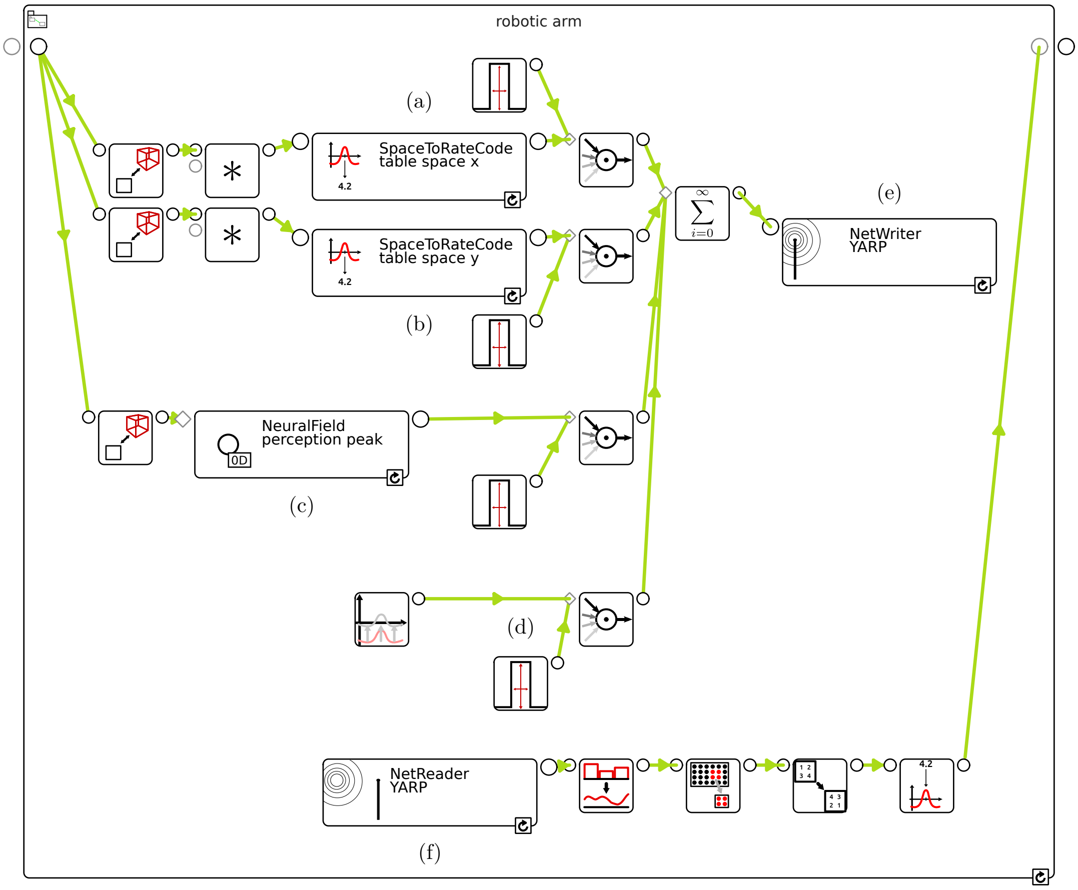

Figure S3. Part of the architecture that implements the connection to the robotic arm. This box shows the insides of the box 'robotic arm' shown in Figure 7 in the main paper.

cedar, and second, because in our lab the camera system and the robotic arm are connected to two different computers. Thus, in this section we will present two parts of the architecture, both of which deal with sensory preprocessing.

We begin with the part that is closest to the camera. Figure S1 shows that part of the architecture as it looks in cedar. The processing step 'camera' at the top left continuously grabs single image frames from the video camera. Each image is sampled down (box (e) to the right of the camera) to reduce the computational load. It is then transformed into the coordinates of the table, resulting in a vertical projection of the image onto the table plane. The transformation is algorithmically computed by the box marked (d), which receives transformation matrices from the box (c), which in turn receives information about the geometry of the table (a) as well as the position, orientation, and internal details of the camera (b).[1] The resulting transformed image is resized again (e) and sent over the network using the YARP framework (Metta et al., 2006).

The second part of the camera preprocessing architecture is hidden in the box labeled 'camera' in Figure 7 in the main article. It is implemented as a group of processing steps, which we have previously collapsed to hide the complexity of that part of the architecture. The inside of the group is shown in Figure S2. The processing step 'NetReader YARP' receives the transformed camera image from the processing step 'NetWriter YARP' shown in Figure S1. The image is then resized (a) to fit the size of the perceptual field

[1] The boxes marked (a), (b), (c), and (d) in Figure S1 are not part of the core of cedar but are added via plugin.

(40×30), the colorspace transformed from BGR (blue, green, red) to HSV (hue, saturation, value) (b), the data format changed from integer to floating point (c), and the HSV channels split into three separate streams (d). Both saturation and value channels (second and third output from the top in box (d)) are thresholded into binary maps (e), which are subsequently summed up (f), and the result divided (h) by 2 (g). This gives us a rudimentary salience map of the image, where highly saturated and bright regions are more salient. The saliency map is combined (i) with the hue channel of the image to form a three-dimensional representation of the data, where the two image dimensions are extended by a hue-dimension. If there is a single salient red object at the top left of the camera image, this representation will have high values at the spatial position of the object and at the color red along the hue dimension; everywhere else, the representation will have low values. This representation is then smoothed with a convolution (j), and the resulting strength of the values adapted by multiplying it with a constant (k). The output of this chain of processing steps leaves the group and feeds directly into the perceptual field.

S1.2 Control of the robotic arm

This section gives more details about the connection of our exemplary architecture to the robotic arm. In our diagram of the complete architecture (Figure 7 in the main article), we did not show the insides of the box ‘robotic arm’, which is a group of processing steps; Figure S3 reveals the inside of that group. The group has two functions: first, it sends the position of the target object to a small program controlling the robotic arm; second, it receives the current position of the end-effector from the same small program and makes it available to the ‘movement CoS field’. We begin with the first function.

As input, the group receives the sigmoided activation of the perceptual field, where the color dimension has already been projected onto the table space. Thus, the input represents the position of the target object within a two-dimensional activation landscape. We project this activation landscape onto the x-dimension of the table, smooth it with a convolution, and extract the position of the peak (see Section 2.5.2 in the main paper). In Figure S3, this is done by the chain of processing steps labeled (a). We do the same for the y-dimension of the table (b). In order to completely stop the arm when there is no peak in the perceptual field, we project its activation onto a zero-dimensional node and use it as a peak detector (c). The node will only be active if there is a peak in the perceptual field. For safety reasons, we added another signal that the user can manually switch on and off and thereby control whether the robot can move (d). These four paths all produce scalar values, which are combined into a single vector with four entries by multiplying them with unit-vectors and summing the results. The vector is then sent to the program controlling the robot arm over the network, using YARP (e).

The second function of the group, reading out the current end-effector position of the arm, is implemented in the chain of processing steps labeled (f). The position is read from the network as a vector. That vector is subsequently converted into floating point notation, the relevant entries are extracted, reordered. Finally, the vector is converted into a two-dimensional space-code representation, where a bump of activation represents the end-effector position of the arm. That activation landscape is then used as the output of the group and fed into the movement CoS field.

The small program that controls the robotic arm and communicates with our architecture over the network is written in C++ using mostly functions that are part of cedar. It consists of a loop that continuously computes the following steps:

1. Read the Cartesian position of the target position from the network using YARP (the values coming from the box in Figure S3e).

2. Send the Cartesian position of the end-effector over the network using YARP (to the box in Figure S3f).
3. Calculate the vector between the end-effector position and the target position.
4. Normalize the length of vector.
5. Scale the vector with a desired, constant speed.
6. Transform the vector to a vector of joint velocities using a Jacobian pseudo-inverse.
7. Send the vector of joint velocities to the hardware and thereby drive the robotic arm.

We are currently working on a generic solution that enables us to implement this kind of program code within the graphical user interface of cedar as well.

REFERENCES

Metta, G., Fitzpatrick, P., and Natale, L. (2006). YARP—Yet Another Robot Platform. *International Journal of Advanced Robotic Systems* 3, 43–48
